# Supplementary material for: Evaluation of B7-H3 Targeted Immunotherapy in a 3D Organoid Model of Craniopharyngioma
Source: Biomolecules. 2022 Nov 24;12(12):1744. doi: 10.3390/biom12121744 (PMC9775874; doi:10.3390/biom12121744)
Supplement: Supplementary file 1 [file biomolecules-12-01744-s001.zip › biomolecules-2025437-supplementary.pdf]

Supplementary Material

1. Supplementary Figures

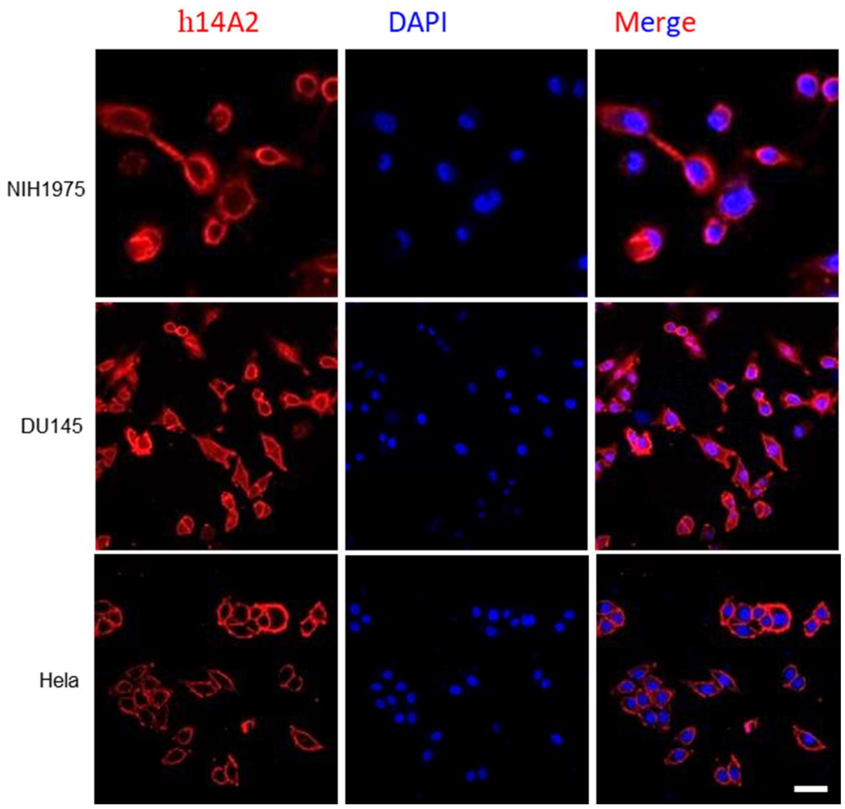

Supplementary Figure S1. Immunofluorescence staining of B7-H3-positive NIH1975, DU145 and Hela cells using monoclonal antibody 14A2. Scale bar: 50 μm

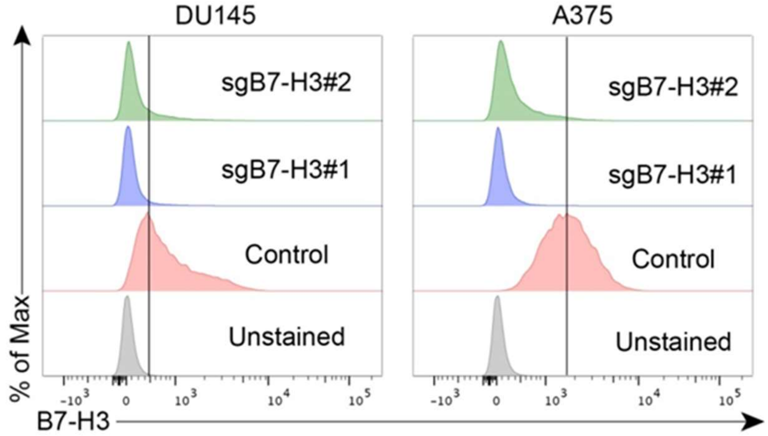

Supplementary Figure S2. Flow cytometry assay for validation of negative expression of B7-H3 in DU145$^{B7\text{-}H3KO}$ and A375 $^{B7\text{-}H3KO}$ stable cells.

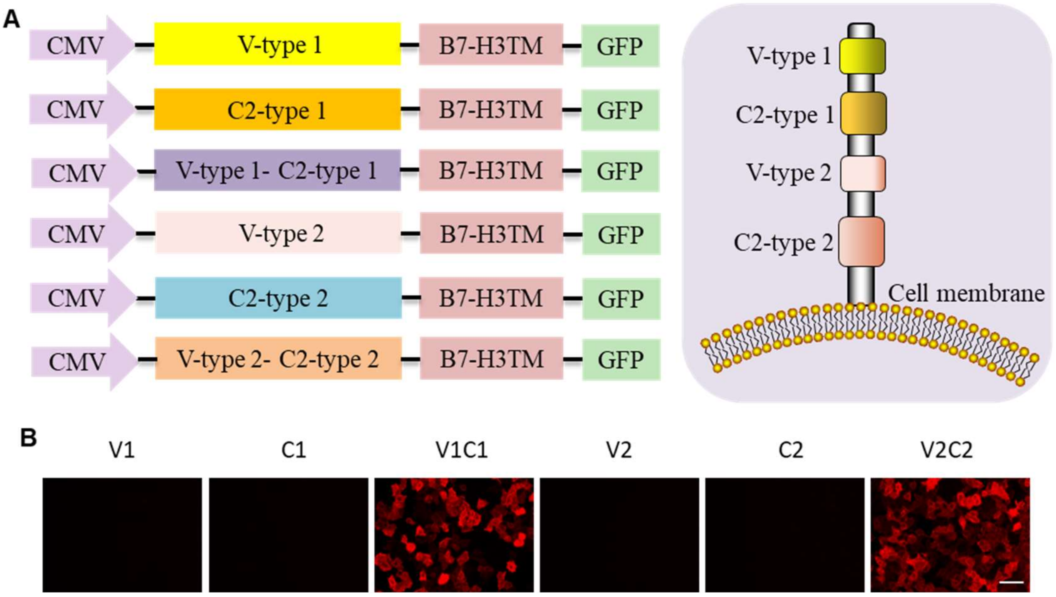

Supplementary Figure S3. Epitope analysis of monoclonal antibody 14A2 binding to B7-H3. (**A**) Schematic diagram of the construction of a lentiviral vector expressing different extracellular domains of B7-H3; (**B**) Immunofluorescence detection of recombinant monoclonal antibody binding to different extracellular domains of B7-H3. Scale bar: 10 μm.

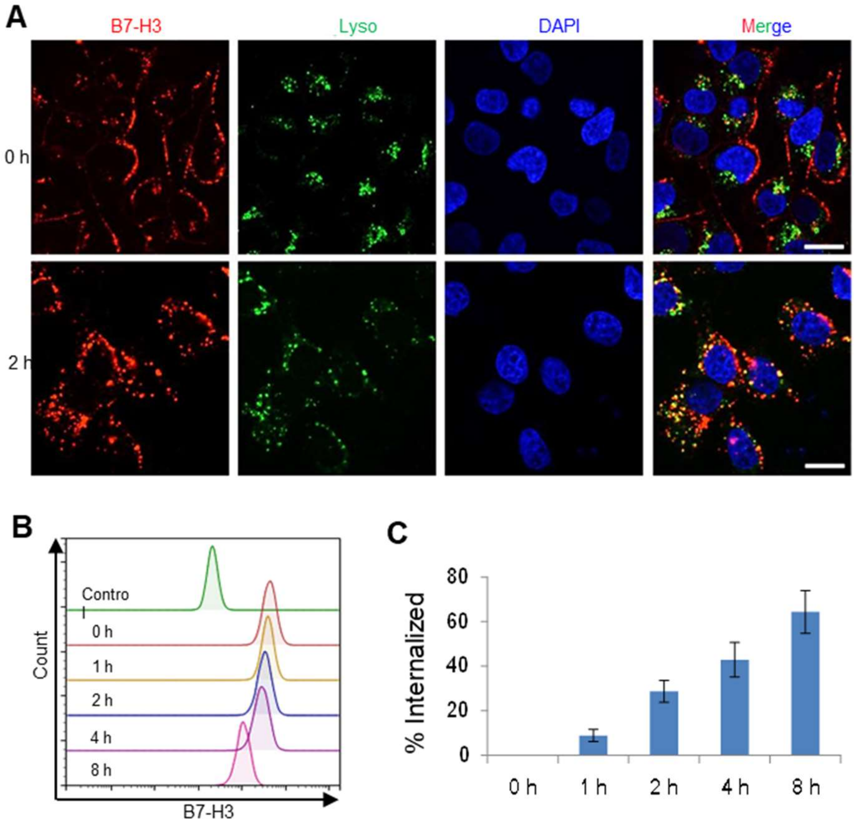

Supplementary Figure S4. Detection of internalization of monoclonal antibody 14A2. (**A**) Immunofluorescence analysis of the endocytic activity of 14A2 antibody in A375 cells. Lyso, lysosome, stained with Lyso-Tracker Green. (**B**, **C**) FACS analysis of the endocytic activity of 14A2 antibody in A375 cells. Experiments were repeated three times, and values were shown as mean ± standard deviation (mean ± SD). Scale bar: 20 μm.
